# High‐resolution multi‐marker DNA metabarcoding reveals sexual dietary differentiation in a bird with minor dimorphism

**DOI:** 10.1002/ece3.6687

**Published:** 2020-09-15

**Authors:** Luís P. da Silva, Vanessa A. Mata, Pedro B. Lopes, Ricardo J. Lopes, Pedro Beja

**Affiliations:** ^1^ CIBIO‐InBIO Research Center in Biodiversity and Genetic Resources University of Porto Vairão Portugal; ^2^ Rua do Torgal nº16 Trigais ‐ Covilhã Erada Portugal; ^3^ CIBIO‐InBIO Research Center in Biodiversity and Genetic Resources Institute of Agronomy University of Lisbon Lisbon Portugal

**Keywords:** bird, diet, high‐throughput sequencing, multi‐marker, *Oenanthe leucura*, resource differentiation

## Abstract

Although sexual dietary differentiation is well known in birds, it is usually linked with significant morphological dimorphism between males and females, with lower differentiation reported in sexually monomorphic or only slightly dimorphic species. However, this may be an artifact of poor taxonomic resolution achieved in most conventional dietary studies, which may be unable to detect subtle intraspecific differentiation in prey consumption. Here, we show the power of multi‐marker metabarcoding to address these issues, focusing on a slightly dimorphic generalist passerine, the black wheatear *Oenanthe leucura*. Using markers from four genomic regions (18S, 16S, COI, and *trn*L), we analyzed fecal droppings collected from 93 adult black wheatears during the breeding season. We found that sexes were rather similar in bill and body features, though males had a slightly thicker bill and longer wings and tail than females. Diet was dominated in both sexes by a very wide range of arthropod species and a few fleshy fruits, but the overall diet diversity was higher for males than females, and there was a much higher frequency of occurrence of ants in female (58%) than male (29%) diets. We hypothesize that the observed sexual differentiation was likely related to females foraging closer to their offspring on abundant prey, while males consumed a wider variety of prey while foraging more widely. Overall, our results suggest that dietary sexual differentiation in birds may be more widespread than recognized at present and that multi‐marker DNA metabarcoding is a particularly powerful tool to unveiling such differences.

## Graphical abstract

We found that males and females of Black wheatears are eating different prey, with males showing a higher prey diversity and females preying more often on ants. These patterns would be undetected with techniques showing lower taxonomic resolution, thus highlighting the potential of metabarcoding in revealing novel ecological patterns.

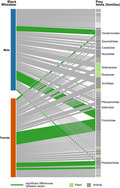

## 1 INTRODUCTION

Sexual partitioning of food resources is known to occur in many animal species, but the extent and ecological significance of this phenomenon are still poorly understood (Ruckstuhl & Neuhaus, 2006). In birds, differences in diet indicative of resource differentiation have mostly been studied in birds with considerable sexual dimorphism in body size (Bravo, Ponce, Bautista, & Alonso, 2016; Catry, Alves, Gill, Gunnarsson, & Granadeiro, 2012; Donals et al., 2007; Gonzalez‐Solis, Croxall, & Wood, 2000; Thalinger, Oehm, Zeisler, Vorhauser, & Traugott, 2018) or in bill size or shape (Smith, 1990; Summers, Smith, Nicoll, & Atkinson, 1990; Temeles, Mazzotta, & Williamson, 2017; Temeles & Roberts, 1993). As a consequence, intraspecific dietary differentiation in birds has been largely attributed to morphological differences, with more sexually dimorphic species expected to show higher resource differentiation (Alarcón et al., 2017; Fonteneau, Paillisson, & Marion, 2009; Lewis et al., 2005; Phillips, McGill, Dawson, & Bearhop, 2011; Selander, 1966). However, it is possible that sexual food resource differentiation also occurs in monomorphic or only slightly dimorphic birds, but this idea remains little explored (but see Botha, Rishworth, Thiebault, Green, & Pistorius, 2017; Cleasby et al., 2015; Elliott, Gaston, & Crump, 2010; Hedd, Montevecchi, Phillips, & Fifield, 2014).

One of the obstacles to understand eventual sexual partitioning of food resources is related to limitations of widely used diet analysis methods, which often are unable to provide enough taxonomic resolution to detect subtle differences in prey consumption (e.g., Mata et al., 2016). This is the case, for instance, of methods widely used in avian ecology, including for instance the morphological identification of the remains of ingested food items (Bravo et al., 2016; Fonteneau et al., 2009; Hunter, 1983; Hunter & Brooke, 1992), direct observation (Catry et al., 2012), fatty acids and alcohols analysis (Owen et al., 2013), or stable isotope analysis (Blanco‐Fontao, Sandercock, Obeso, McNew, & Quevedo, 2013; Cleasby et al., 2015; Elliott et al., 2010; Hsu, Shaner, Chang, Ke, & Kao, 2014; Ludynia et al., 2013; Paiva et al., 2018; Phillips et al., 2011). The advent of high‐throughput DNA sequencing is making it possible to overcome the limitations of these methods, providing the ability to identify virtually all prey species consumed with unprecedent taxonomic resolution (Hope et al., 2014; Nielsen, Clare, Hayden, Brett, & Kratina, 2017; Razgour et al., 2011; Soininen et al., 2009). As a consequence, this approach has been increasingly used to describe the diets of a wide range of animals (Brown, Jarman, & Symondson, 2012; Kaunisto, Roslin, Sääksjärvi, & Vesterinen, 2017; Macías‐Hernández et al., 2018; Mata et al., 2016; Soininen et al., 2009), including birds (Coghlan et al., 2013; Deagle, Chiaradia, McInnes, & Jarman, 2010; Jedlicka, Vo, & Almeida, 2017; Liu et al., 2018; Sullins et al., 2018; Trevelline et al., 2018). The high taxonomic resolution provided by high‐throughput sequencing has already been used to describe sexual dietary differences that otherwise would be almost impossible to detect (Mata et al., 2016). However, previous studies have focused on specialists with a relatively narrow feeding niche, while this methodology remains underexplored in testing sexual dietary in more generalist species such as many omnivorous passerines. Dietary generalists are more challenging to study using metabarcoding because they require a combination of markers to fully encompass the full spectrum of food resources used (da Silva et al., 2019a).

Here, we aim to show the power of multi‐marker metabarcoding to investigate differences in diet between sexes, by focusing on a generalist passerine judged to have minimal sexual dimorphism, the black wheatear (*Oenanthe leucura*). To address this general goal, the study first documents difference in morphology (bill and body features) between sexes and then use a previously developed approach for integrating metabarcoding dietary data across multiple markers (da Silva et al., 2019a) to describe the diets of both sexes. Using this data, we then tested the hypothesis that diet varies between sexes in terms of (a) diet diversity and (b) frequency of occurrence of the main food items and that (c) sexual dietary differentiation can only be detected at the high taxonomic resolution provided by metabarcoding. Results were used to discuss the potential of multi‐marker metabarcoding to provide a detailed understanding of intraspecific variation in bird diets.

## 2 MATERIAL AND METHODS

### 2.1 Study area and species

The study was conducted in northeast Portugal, along the Douro river valley and surrounding areas, which corresponds to the last stronghold of the black wheatear in the country. This population occurs mainly in traditional vineyards and olive groves (terraces with stone walls) and is spatially isolated (>100 km) from the remaining Iberian population.

The black wheatear is a highly territorial passerine that occurs in arid and semiarid regions of the Iberian Peninsula and North Africa. Although the species is not globally threatened, European populations are declining, and the species is now considered regionally Vulnerable in Europe (BirdLife International, 2015) and Critically Endangered in Portugal (Cabral et al., 2005). It is a sedentary species, considered to have a monogamous mating system and likely a lifelong pair‐bond, with eggs being incubated only by the female and offspring being fed by both parents (Richardson, 1965). Previous studies using conventional morphological approaches have shown that the species feeds on a wide range of animal and plant food items, none showing any sexual dietary differences (Hodar, 1995; Múrias, Ribeiro, Nunes, & Gomes, 2008; Prodon, 1985; Richardson, 1965).

### 2.2 Field sampling

To document the morphology and diet of black wheatears, we carried out captures throughout the study area, during the entire breeding season from April to August of 2014 to 2016, using spring traps baited with mealworms (*Tenebrio molitor*). Birds were removed from the traps immediately after being captured, placed in a cotton bag, and afterward ringed and measured. Birds were retained for less than 15 min, and all procedures were made with the required permits from national authorities. We made a total of 143 captures, but for this study, we only considered the first capture of adult individuals, that is, 2nd calendar year or more identified following Svensson (1992), totaling 110 adult black wheatears, 79 males and 31 females. For each individual, a number of morphometric measures were taken following Svensson (1992): maximum cord wing length; 3rd primary length; tail length; tarsus length; bill length, depth, and width at the distal edge of the nostril; and body mass. Wing, 3rd primary and tail were measured using a ruler to the nearest 0.5 mm, tarsus and bill measurements were made with a calliper to the nearest 0.1 mm, and body mass with a digital balance to the nearest 0.1 g. All measures were taken by LPS, and when feathers were not fully developed (i.e., molting birds), the measurements affected were not recorded (Table S1).

Droppings for molecular analysis were collected from bird handling bags or directly from small rocks used to disguise the bottom of the spring traps (McInnes et al., 2017; Oehm, Juen, Nagiller, Neuhauser, & Traugott, 2011). Bags were soaked in 10% bleach for 1 hr and then washed between each use to minimize contamination. From the 93 droppings thus collected, 62 from males and 31 from females, three were obtained from birds that defecated inside the traps but were not captured. Droppings were stored in 2‐ml tubes with 98% ethanol at 4°C until laboratory analysis (da Silva et al., 2019a).

### 2.3 Diet analysis

The current study examines the diet of black wheatears based on a subset of data generated by da Silva et al. (2019a), who evaluated the limitations and biases associated with single marker metabarcoding in the dietary analysis of trophic generalists and proposed a multi‐marker approach to provide a more comprehensive description of species' diets. The current study furthers the previous work of Silva et al. (2019a), using their methodology to explore differences in diet between males and females of the same species. This way, we selected 93 droppings collected upon the first capture of 110 adult birds, thereby avoiding biases that might result from including data from birds captured more than once (pseudoreplication), as well as eventual confounding effects of including a small number of 1st calendar year birds. Laboratory and bioinformatic procedures are described in detail in da Silva et al. (2019a). Briefly, the DNA of the droppings was extracted in batches of 23 samples plus a negative control, using the Stool DNA Isolation Kit (Norgen Biotek Corporation) and following the manufacturer's protocol. DNA extracts were then subjected to four independent PCR reactions, each targeting complementary taxa and different gene regions: 18S (Jarman et al., 2013) for eukaryotes; 16S (da Silva et al., 2019a) and COI (Zeale, Butlin, Barker, Lees, & Jones, 2011) for arthropods; and *trn*L (Taberlet et al., 2007) for plants. These regions and primers were selected to maximize the detection of all the expected food items of black wheatears (Hodar, 1995; Múrias et al., 2008; Prodon, 1985; Richardson, 1965). PCR products were diluted 1:4 and amplified again to incorporate Illumina indexes. Resulting fragments were purified using AmPure Beads, quantified in NanoDrop, normalized, and pooled per primer. Each library was further quantified using qPCR, normalized to 4 nM, and pooled. The final pooled library was sequenced in an Illumina MiSeq using a partial V2 2x250bp kit with an expected sequence coverage of 12,000 reads/primer/sample (see Table S1 for detailed number of raw reads obtained per sample). Bioinformatic procedures were done using 'ObiTools' and consisted in pairwise alignment of reads, removal of primer sequences, collapsing of reads into exact sequence variants (ESVs), and removal of nontarget and potential spurious sequences using 'obigrep' and 'obiclean'. PCRs with less than 100 reads after these steps were considered as having failed and were removed from further analyses. This happened with all negative controls and a few taxa specific PCRs of some samples (16S, COI, and *trn*L). Finally, ESVs that had a read count <1% of the total number of reads of each PCR were removed and the remaining ones were assigned to a prey item by blasting each ESV against BOLD and NCBI online databases and COI sequences from arthropods collected in Portugal (Ferreira et al., 2018, 2020). Each possible taxon was checked for its occurrence in the Iberian Peninsula and discarded if not known to occur in either Portugal or Spain. Species‐level identifications were usually made at identity levels above 98.5% with a single species, except for rare cases where no other species of the genus were known to exist in Portugal. If the same ESV matched different species, genus, or families, identifications were made to the lowest taxonomic level possible that encompassed all the closest hits. Whenever different ESVs matched the same taxa or groups of sequences, they were joined into a single molecular unit. Each molecular unit was then given a unique identifier based on the most resolved taxonomic assignment possible. For example, if two units were only possible to identify to the family level (e.g., Carabidae), one was named Carabidae 1 and the other Carabidae 2. In some cases, the name given to molecular units refer to taxonomic subgroups between order and family or family and genus, if such identification was possible. We assumed that each molecular unit potentially corresponded to a different species, although many of those units were not possible to identify to the species level due to lack of reference sequences.

For diet analysis, we only considered molecular units identified at least to the order level and excluded all taxa that were considered unlikely to be eaten by wheatears (see Table S1 for detailed number of reads retained after biodinformatic and taxa filters per sample). We therefore excluded all items that were likely sampling contaminations (e.g., human, fungi, and mealworm DNA), and other items not likely to be intentionally ingested by wheatears such as bird parasites. Regarding plants, we retained only those with ripe fleshy fruits that were likely to be eaten by birds, assuming that all other plant occurrences resulted from secondary ingestion through the stomach contents of herbivore arthropods. This was considered a reasonable assumption, because previous studies suggested that black wheatears eat fleshy fruits but not other plant parts such as leaves or dry seeds (Hodar, 1995; Múrias et al., 2008; Prodon, 1985; Richardson, 1965), and this view was confirmed by the parallel morphological and molecular examination of fecal samples undertaken by da (Silva et al., 2019a). Following the identification per marker, we integrated all the dietary items recovered across the four molecular markers into a single dataset (Table S2) using the Python script provided by da Silva et al. (2019a). This script merges the redundant taxa found with the different primers, this way creating a dataset composed by distinct taxa, or operational taxonomic units (OTUs).

### 2.4 Data analysis

All statistical analysis was performed in R v3.5.2 (R Core Team, 2018). A significance level of *α* = .05 was considered. To test for sexual size dimorphism, we compared all the adult bird's measurements (wing, 3rd primary, tail, tarsus, weight, bill length, depth, and width) with a multivariate ANOVA using the R's base function “*MANOVA*.” To understand which of these measurements mostly contributed to differences, we further performed univariate tests using the function “*summary.aov*.” Dietary analysis was based on the presence/absence of taxa per dropping analyzed, considering 3 different taxonomic levels: OTU (all taxonomic units identified to the most possibly resolved taxonomic level, even if the unit was classified only up to family or order level), family and order. We used OTUs as the most resolved taxonomy instead of species, because many taxa could not be identified to that level due to gaps in reference databases, and because excluding taxa identified only at higher taxonomic levels would bias results because reference collections are highly unbalanced across taxonomic groups. To compare the average number of prey taxa detected per dropping of males and females, we built a GLM with a Poisson error distribution using the R's base function “*glm”* and tested its significance using the function “*ANOVA”* of the package “car” (Fox & Weisberg, 2011). The overall richness of prey ingested by both sexes was estimated using Hill numbers with the double of the reference sample size to avoid extrapolation bias (Chao et al., 2014). For this, we used the function “*iNEXT”* of the package “iNEXT.” We compared the estimated richness considering completeness, that is, sample coverage, instead of sample size, that is, number of samples, to avoid biases of communities with different levels of richness requiring different sampling efforts in order to be sufficiently characterized (Chao & Jost, 2012). Instead of comparing the 95% confidence interval, a very conservative approach, we considered that differences were significant if the 84% confidence interval (a proxy for *α* = .05) of both estimates did not overlap (MacGregor‐Fors & Payton, 2013). Finally, we compared diet composition between sexes by building a generalized linear model for multivariate abundance data with a binomial distribution using the function “*manyglm”* from the package *“*mvabund” (Wang, Naumann, Eddelbuettel, & Warton, 2018) and tested for its significance with the function “*anova.manyglm”* of the same package. As there were no significant differences between males and females in sampling day (GLM with negative binomial distribution: LR Chisq = 1.066, *df* = 1, *p* = .302), latitude (GLM with Poisson distribution: LR Chisq = 2.149, *df* = 1, *p* = .143) and longitude (GLM with negative binomial distribution: LR Chisq = 2.056, *df* = 1, *p* = .152) of sampling sites, they were not considered in models as potentially confounding variables. To further assess which prey items were responsible for differences in diet between both sexes, we looked at the *p*‐values of univariate tests outputted by the function “*anova.manyglm*.”

## 3 RESULTS

### 3.1 Morphology

Black wheatears showed significant sexual dimorphism in the studied measurements (MANOVA: Pillai's trace = 0.502, *F*_1,91_ = 10.594, *p* < .001). The univariate tests showed that females had on average shorter wing (4% difference), 3rd primary (5%) and tail (2%), as well as a thinner bill (2%), while the other measurements (tarsus, body mass, bill length, and width) were not significantly different between sexes (Table 1).

**TABLE 1:** Biometric differences between adult black wheatear sexes

Measurement	Female	Male	Univariate test
Wing	95.318 ± 0.976	99.648 ± 0.479	***F* = 73.756, *p* < .001**
3rd primary	70.955 ± 0.783	74.514 ± 0.463	***F* = 58.078, *p* < .001**
Tail	68.595 ± 0.917	69.824 ± 0.489	***F* = 9.182, *p* = .003**
Tarsus	27.159 ± 0.387	27.215 ± 0.216	*F* = 0.065, *p* = .799
Body mass	34.875 ± 1.274	35.531 ± 0.502	*F* = 1.344, *p* = .249
Bill length	12.659 ± 0.330	12.928 ± 0.159	*F* = 2.554, *p* = .114
Bill width	4.232 ± 0.086	4.285 ± 0.061	*F* = 0.783, *p* = .379
Bill depth	4.409 ± 0.092	4.517 ± 0.048	***F* = 4.653, *p* = .034**

Note

All measures are in mm except body mass that is in grams. Average ± 95% confidence interval and MANOVA univariate tests (*F* and *p* value). Significant values are in bold.

### 3.2 Diet

**FIGURE 1:**
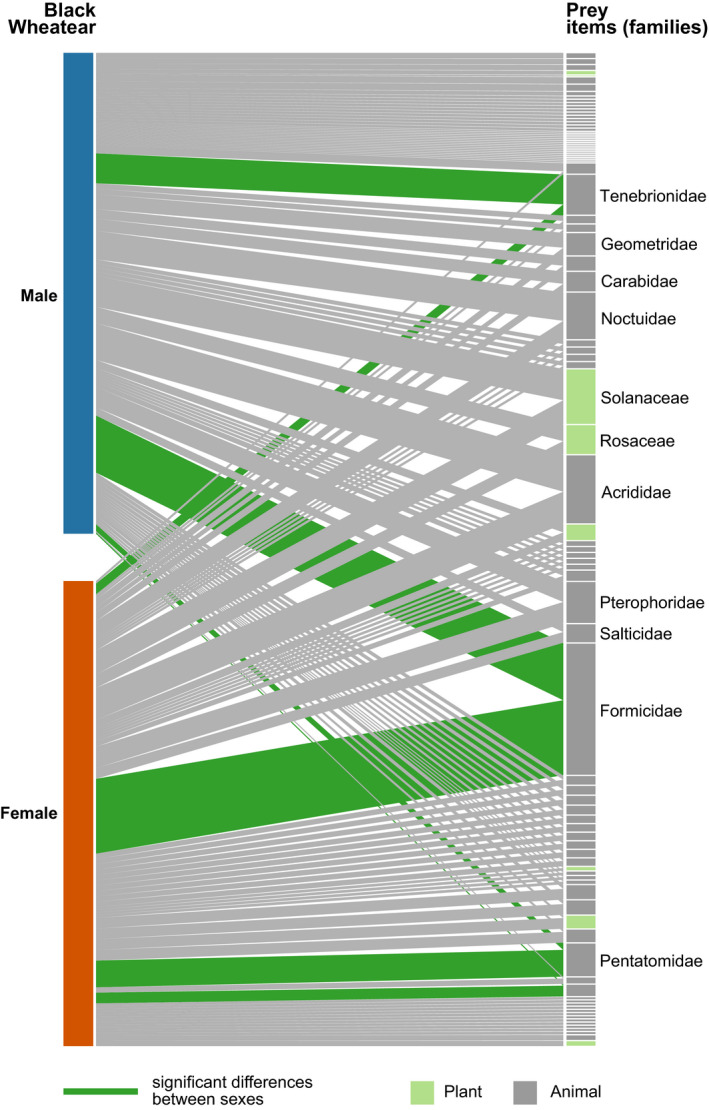
Frequency of occurrence network showing the families ingested by black wheatear males and females. On the right, animal families are in gray and plant families in light green. Dark green interactions indicate families consumed in significantly different proportions by both sexes, as revealed by univariate tests. Only the names of the most frequent families (more than 10% frequency) are shown

The diet of black wheatears was very diverse, with 338 OTUs of 96 families, 29 orders (Table S3), and 7 classes (Magnoliopsida, Reptilia and 5 Arthropoda classes). Arthropods were detected in all samples and belonged to 22 orders, of which 17 orders were Insecta. The main prey belonged to the order Hymenoptera (Frequency of Occurrence: 83%), mainly ants (family Formicidae; 75%). Frequent arthropod orders that were detected in more than half of the samples included Lepidoptera (67%), mainly belonging to families Noctuidae (30%), Pterophoridae (25%), and Geometridae (15%); Coleoptera (62%), mainly Tenebrionidae (28%) and Carabidae (13%); Orthoptera (54%), mainly Acrididae (42%); and Diptera (51%), with 10 families identified but none detected in more than 10% of droppings. There were also other important arthropods as Hemiptera (40%), mainly from the family Pentatomidae (16%); and Araneae (34%), mainly Salticidae (11%). The only vertebrates found were lizards (Squamata) detected in two droppings. Considering only plants judged to be consumed directly by wheatears, the plant component of the diet was less diverse, but also very common (60% of the droppings), with *Solanum nigrum* (order Solanales, family Solanaceae) being the most frequently detected (35%) (Figure 1; Table S3).

**FIGURE 2:**
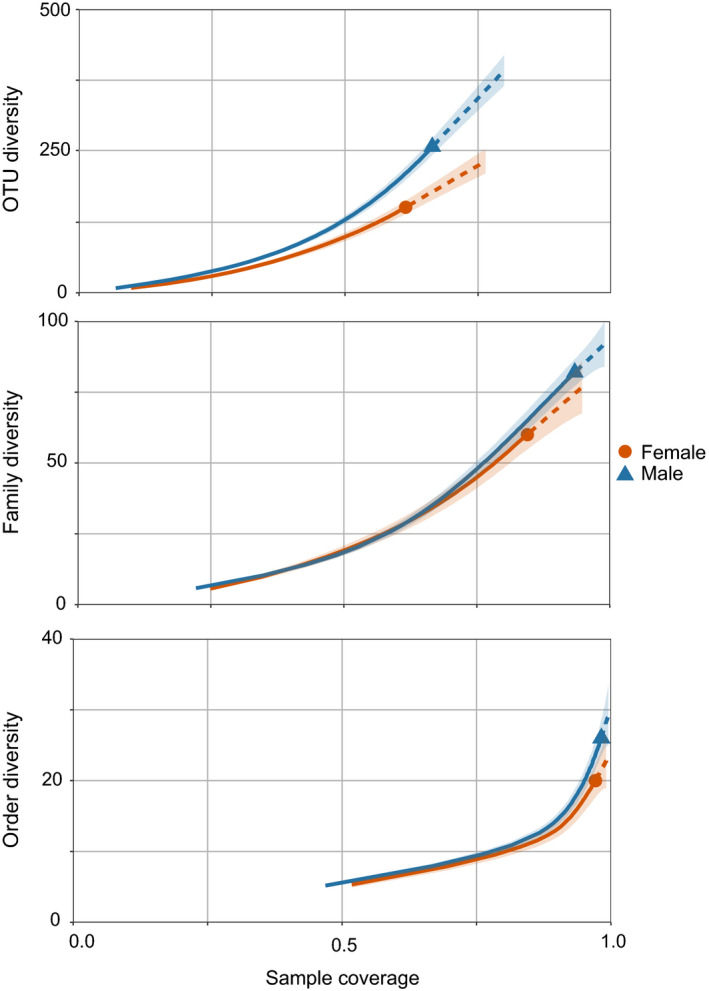
Rarefaction curves showing the observed (full line) and estimated (dashed line) richness, until double the reference sample size, and respective 84% confidence interval (a proxy for *α* = .05) by sample coverage

We found no differences between sexes in the average number of prey items detected per sample, irrespective of taxonomic resolution: OTU ($\bar{x}$ = 8.398; LR Chisq = 0.430, *df* = 1, *p* = .512), families ($\bar{x}$ = 5.739; LR Chisq = 0.130, *df* = 1, *p* = .718), or orders ($\bar{x}$ = 5.226; LR Chisq = 0.083, *df* = 1, *p* = .773). However, the analysis based on sampling completeness indicated that the overall prey richness was higher for males than females at the OTU level (even if a 95% confidence interval was considered), while no significant differences between sexes were detected at the family or order levels (Figure 2).

Regarding diet composition, we found a significant difference between sexes at the OTU (Res. *df* = 91, Deviance = 430.1, *p* = .010) and family levels (Res. *df* = 90, Deviance = 139.9, *p* = .021), but not at the order level (Res. *df* = 91, Deviance = 44.52, *p* = .054). The univariate tests showed that the differences were due to 10 OTUs and 6 families (Table S3). The prey item most important for compositional differences was one unidentified Myrmicinae species that was also the prey most often detected in black wheatear droppings. This ant species was detected in 58% of females' droppings, while its frequency of occurrence in males was just 29% (Table S3). At the OTU level, all other prey had differences in frequency of occurrence between sexes smaller than 10% (Table S3). At the family level, the differences were mainly due to families Pentatomidae, Formicidae, and Tettigoniidae, that were preyed 24%, 21%, and 11%, respectively, more often by females, while males preyed 23% more often on Tenebrionidae (Figure 1; Table S3). There were also 2 orders that differed between sexes (Hymenoptera and Santales), despite the overall effect of sex being nonsignificant when analyzing prey composition at the order level (Table S3).

## 4 DISCUSSION

Our results supported all our hypothesis, showing that although black wheatears exhibit only minor sexual size dimorphism there was dietary differentiation between both sexes, by (a) males having an overall higher diet diversity and (b) females preying more often on some ant species than males. Moreover, results supported the idea that (c) the detection of differences between sexes were conditional on the high taxonomic resolution provided by metabarcoding.

In fact, the differences found in diet richness and composition were smaller or not significant using higher taxonomic ranks (order or family), suggesting that if methodologies yielding lower taxonomic resolution had been used, these differences would not have been detected. This is the first time differences between sexes of bird species with minor sexual dimorphism are either studied or found in birds using metabarcoding, underlining the power of these techniques in bird ecology. This methodology could be particularly relevant for birds such as passerines and near passerines, that feed on hyperdiverse taxonomic groups that are often difficult to identify, as insects and other arthropods, and in which diets are often evaluated to the order or family level through conventional techniques (Araújo, Lopes, da Silva, & Ramos, 2016; Catry et al., 2019; Hodar, 1995). Moreover, metabarcoding, and other DNA‐based methods, can improve the detection of many prey items, specially soft‐bodied items as lepidoptera, thus advancing the knowledge of the overall diet of organisms (Nielsen et al., 2017; da Silva et al., 2019a).

The morphometric differences between sexes observed in our study were related to the thicker bill and longer wings and tail of males. In previous studies conducted in Alicante (Pérez‐Granados & Seoane, 2018) and Hoya de Guadix (Møller, Lindén, Soler, Soler, & Moreno, 1995), Spain, males were described not only as having longer wings (wing length and 3rd primary) and tail, but also as being heavier and with a longer tarsus than females. This indicates that sexual size dimorphism on this species may differ across its distribution. Reasons for this are uncertain, but it may be a consequence of for instance local adaptation in a small and isolated (>100 km) population such as ours, or to low densities eventually reducing competition between males, though this hypothesis would need to be tested. The fact that our males showed longer wings and tail, but similar body mass and tarsus, a proxy for body size (Freeman & Jackson, 1990; Pérez‐Granados & Seoane, 2018; Rising & Somers, 1989), suggests a higher flight capability of males compared to females. It has been suggested that the larger wings and tail of male black wheatear's could be related to their stone‐carrying behavior (Pérez‐Granados & Seoane, 2018; Soler, Soler, Møller, Moreno, & Lindén, 1996) that is mainly done by males (Aznar & Ibáñez‐Agulleiro, 2016; Moreno, Soler, Møller, & Linden, 1994). Males also move more often in their territories than females, especially for territory defence, not only against conspecifics, but also against other birds of different sizes (Møller, 1992; Prodon, 1985). Regarding the thicker bill of males, it could also be an adaptation to the stone‐carrying behavior and higher aggressivity.

The dietary composition of black wheatear observed in our study was largely similar to that documented elsewhere. In particular, the large dietary spectrum of arthropod groups and the ability to hunt relatively large prey such as reptiles was already reported from natural habitats of Spain, where the most frequent prey were also ants (Hodar, 1995; Richardson, 1965). The highest difference found between previous dietary studies of this species and our work is the high frequency of berries detected in our study. To some extent, this could be due to the different methods used for the identification of the droppings remains (da Silva et al., 2019a). However, it is more likely related to differences in habitat, since the Portuguese population occurs mainly in traditional agricultural habitats (vineyards and olive groves) where *Solanum nigrum* is a very widespread and abundant herb, providing a high number of ripe fruits. In contrast, the studied Spanish populations were located in shrub‐steppe areas, presumably with a lower availability of berry‐bearing plant species during the wheatear's breeding season (Hodar, 1995).

The sexual differences in diet composition observed in our study are likely more related to behavioral differences during the breeding season than to morphometric differences between males and females. Although males have a more robust bill than females, its length and width are similar, which in principle allows both sexes to capture and swallow similar prey items. In some birds, it has been reported that females tend to forage closer to their offspring than males (Sunde, Bølstad, & Møller, 2003). This behavior could lead females to feed more often on abundant and predictable prey like ants, even if these are smaller and less nutritious (Dean & Milton, 2018). On the other hand, the higher mobility of males within territories could explain the lower frequency of some less nutritious prey (e.g., ants), and the wider range of other prey, likely less predictable and abundant.

To the best of our knowledge, this is the first example of a monomorphic (or minor dimorphic) passerine species exhibiting dietary differences between sexes, during the breeding season. Usually, the more sexually dimorphic a bird species is, the higher resource differentiation is expected (Fonteneau et al., 2009; Lewis et al., 2005; Phillips et al., 2011; Selander, 1966). Nevertheless, on some monomorphic seabirds species, different foraging areas have been described between sexes, especially in the beginning of the breeding period (Cleasby et al., 2015; Hedd et al., 2014; Pinet, Jaquemet, Phillips, & Le Corre, 2012). On two New Guinean whistlers, passerine species with little sexual dimorphism, vertical segregation was also found between sexes and attributed to male territory defence and intersexual food resource differentiation (Freeman, 2014). Nonetheless, it is not clear how spatial segregation translates into dietary segregation, and there seems to be little evidence of dietary segregation in monomorphic species (Catry et al., 2019; Phillips et al., 2011), despite some exceptions (Cleasby et al., 2015). Regardless of the main cause for the dietary differentiation found in our study, it shows a sexual dietary differentiation during the breeding period, which may help lowering intraspecific competition, which can be especially important in the (semi‐)arid landscapes where black wheatears occur.

Overall, our study shows how even minor dimorphic bird species can have subtle differences in diet during their breeding season. The differences found were most likely related to sexual differences in behavior rather than morphology, which means that this pattern might be far more common than what is currently recognized in birds. Moreover, this pattern was only possible to detect thanks to the high taxonomic resolution offered by metabarcoding, as analyses at higher taxonomic ranks were not able to identify such differences. At a time when metabarcoding is starting to be used to revisit and assess the diet of many species, as well as to study other species interactions like pollination, it becomes increasingly important to understand the impact of taxonomic resolution in ecological studies, although species‐level identifications may not always be necessary depending on the study objectives (Renaud, Baudry, & Bessa‐Gomes, 2020). Finally, this study is an example of how the development of new techniques, such as metabarcoding, can help ecological studies go a bit further and gain better insights into fine ecological patterns that could otherwise go unnoticed.

## CONFLICT OF INTEREST

The authors have no conflict of interest to declare.

## AUTHOR CONTRIBUTIONS

**Luís P. da Silva:** Conceptualization (lead); data curation (lead); formal analysis (lead); funding acquisition (supporting); writing – original draft (lead); writing – review & editing (equal). **Vanessa A. Mata:** Methodology (equal); validation (equal); visualization (equal); writing – review & editing (equal). **Pedro B. Lopes:** Data curation (equal); validation (equal); writing – review & editing (equal). **Ricardo J. Lopes:** Validation (equal); writing – review & editing (equal). **Pedro Beja:** Funding acquisition (lead); validation (equal); writing – review & editing (lead).

## Supporting information

Supplementary Material

## Data Availability

Morphometric and dietary matrices used for analysis are available in supporting information (Tables S1 and S2). Raw sequencing data are available at Dryad: https://doi.org/10.5061/dryad.26vr077 (da Silva et al., 2019b).
